# Critical Path for Alzheimer’s Disease (CPAD) Consortium: Data‐Driven Solutions for Clinical Trial Design and Informed Decision Making

**DOI:** 10.1002/alz.086145

**Published:** 2025-01-09

**Authors:** Sudhir Sivakumaran, Eileen Priest, Corissa Lau, Michael C. Irizarry, Yashmin Karten

**Affiliations:** ^1^ Critical Path Institute, Tucson, AZ USA; ^2^ Eisai Inc., Nutley, NJ USA; ^3^ Critical Path for Alzheimer’s Disease (CPAD) Consortium, Critical Path institute, Tucson, AZ USA

## Abstract

**Background:**

To help improve the Alzheimer’s disease (AD) therapeutics research and development process, the Critical Path for Alzheimer’s Disease (CPAD) Consortium at the Critical Path Institute (C‐Path) provides a neutral framework for the drug development industry, regulatory agencies, academia, and patient advocacy organizations to collaborate. CPAD’s extensive track record of developing regulatory‐grade quantitative drug development tools motivates sponsors to share patient‐level data and neuroimages from clinical trials. CPAD leverages these data and uses C‐Path’s core competencies in data management and standardization, quantitative modeling, and regulatory science to develop tools that help de‐risk decision making in AD drug development.

**Method:**

Clinical data are curated, standardized, and aggregated into analysis subsets, which are used to develop disease progression models that form the basis for clinical trial simulation (CTS) tools. Such tools help optimize patient and endpoint selection, and the design of efficacy studies. CPAD’s database includes raw neuroimages from contemporary datasets, which are used to lead two pre‐competitive efforts, in collaboration with leading academic and industry experts, to 1) test and validate a tau‐PET quantification method that harmonizes derived measures across tracers and cohorts, and 2) explore and evaluate the readiness of tau‐PET as a surrogate marker in AD drug development to support accelerated drug approval.

**Result:**

As of November 2023, CPAD’s clinical trial repository contains 73 studies with over 100,000 individual de‐identified patient records, with a rich source of key AD biomarkers (biofluids and imaging). Different linear and non‐linear mixed effects models have been fit based on relevant biomarker combinations, characterizing the time course of clinically relevant outcome measures. To harmonize tau‐PET quantification, two different methods (a Centiloid‐like approach and a Joint Propagation model) were tested and validated across multiple tracers, cohorts and studies. A data and research plan has been drafted to explore and evaluate the readiness of tau‐PET as a surrogate marker in AD.

**Conclusion:**

The precompetitive collaboration by CPAD is fundamental to the generation of actionable quantitative drug development tools for accelerating and advancing AD drug development and building consensus among stakeholders that provide confidence to sponsors for the adoption of the tools.

